# Niagara University

**Published:** 1890-05

**Authors:** 


BUFFALO MEDICAL AND SURGICAL JOURNAL

A MONTHLY REVIEW OF MEDICINE AND SURGERY.

*EDITORS:*

THOS. LOTHROP, M. D. - - - W. W. POTTER, M. D.

All communications, whether of a literary or business character, should be addressed to the editors: 284 FRANKLIN STREET, BUFFALO, N. Y.

### Editorial.

### NIAGARA UNIVERSITY.

### MEDICAL DEPARTMENT.—ANNUAL COMMENCEMENT.

AMONG the events of scientific and professional interest occurring in this city during the month just closed, we are gratified to note the exercises attending the close of the college year of this institution. Rarely, if ever before, has the profession here enjoyed such a treat. The character and ability of the men engaged in the discussions gave a tone to its public exercises and proceedings which demonstrates the influence of this school of medicine in the community, and the sagacity with which its interests are managed. We may also refer to the fact that when medical men near at home and from distant cities grace with their presence an occasion of this kind, they are giving a quasi-endorsement to the principles on which this school was founded, and also affording encouragement to the projectors of the movement in the interests of higher medical education, which will be felt by an advance all along the line in medical schools throughout the country.

The grade examinations and also the examinations for the degree continued two weeks, and in the wide range of subjects and severity of the tests required, exceeded any trial to which the students had been previously subjected. A high standard has been aimed at and fully maintained the present year.

The alumni meeting was held at the college, and the address of welcome was delivered by Prof. Herbert Mickle, after which the President, Dr. Geo. W. T. Lewis, gave the annual address. The following officers were duly elected: *President*, David L. Redmond, M. D.; *1st Vice-President*, John J. Twohey, M. D.; *2d Vice-President*, A. W. Bayliss, M. D.; *Secretary*, Edgar A. Forsyth, M. D.; *Permanent Secretary*, Frank H. Potter, M. D.; *Treasurer*, H. D. Ingraham, M. D.; *Executive Committee*, J. M. Hewitt, M. D., E. J. Murphy, M. D., Sydney A. Dunham, M. D.

In the afternoon the alumni met for the reading and discussion of papers of scientific and professional interest.

Dr. W. S. Tremaine gave some valuable remarks on The Use of Electricity in Myomata of the Uterus.

Dr. Joseph Hoffman, of Philadelphia, read a paper on The Treatment of Lesions at and about the Head of the Colon.

Prof. Burt G. Wilder, M. D., of Cornell University, gave a résumé of the present knowledge on The Sub-Frontal Gyre (Broca's convolution) in Man and Apes.

It need not be here stated that these subjects were ably presented, and elicited a discussion from those present which fittingly supplemented the labors and opinions of the essayists.

Where all the papers were of such a high order of scientific value, it may be invidious to particularize, but we think it due to Prof. Wilder to state that, as his subject was out of the order of those usually presented at meetings of this kind, the familiarity of the speaker with the anatomy of the brain, and the clearness with which the matter was presented, demonstrated the wide range of knowledge of the essayist and his eminent ability in this special theme. A special interest, however, centered in the electrical discussion that followed Dr. Tremaine's paper. Much of the atmospheric fogginess that has enshrouded the therapeutic uses of electricity in Buffalo was cleared away, and all seemed to feel better after hearing the logical setting forth of its status by the debaters.

The exercises of the evening were held at the Star Theatre, and consisted of the conferring of the degree of Doctor of Medicine on the following young men, who had successfully passed the ordeal of examination by the Faculty, and subsequently by the Board of Medical Examiners. The names are arranged in the order of standing: James Henry Dowd, Batavia; Bentley Silas Bourne, Hamburg; Edward Michael Dooley, Meriden, Conn.; Linus James McAdams, Buffalo; Charles John Reynolds, East Otto; Albert J. Colton, West Webster; Matthew Joseph O'Connell, Trumansburg; James P. Touhey, Monterey; Edwin Andrew Millring, Buffalo; William Henry Norrish, Buffalo; James Joseph Mooney, Buffalo; Henry Lovell Gillette, Batavia; Nicholas Lawrence Mulvey, Fairmount; Christopher Morris Kelley, Jr., Ansonia, Conn.; William Rodney Griffis, Buffalo.

We regret to state that the Chancellor of the University, Rt. Rev. Stephen V. Ryan, D. D., was not present on account of ill-health, but his place was filled by Rev. P. V. Kavanagh, Vice-Chancellor.

Prof. Simeon Tucker Clark, M. D., delivered a poem of rare merit on Science and Faith. It is due to the speaker to state that he filled, on a short notice, the gap occasioned by the failure of Rev.

Henry A. Adams to be present, whose regrets were made, and reluctantly accepted by all present.

The address to the graduates was given by Dr. Lewis S. McMurtry, of Louisville, Ky. A synopsis of this admirable production would do injustice to its merits, and we must be content to state that in beauty of diction, and in conciseness and clearness of statement, Dr. McMurtry demonstrated that his pen was controlled by a master hand, and inspired also by superior mental gifts. We trust this address will be printed in full.

The banquet in the evening at the Genesee concluded the exercises, and gave the opportunity for those who enjoy a "feast of reason and a flow of soul," to satisfy themselves to the fullest extent. Dr. F. H. Potter performed the duties of toast-master with all necessary grace and propriety. The regular toasts and the gentlemen responding to them, were as follows: "The State of Kentucky," Dr. Lewis S. McMurtry, of Louisville; "The Quaker City," Dr. Joseph Hoffman, of Philadelphia; "The City of Buffalo," Philip A. Laing; "The Press," F. J. Shepard of the *Courier*; "Our Manufacturing Interests," J. B. Olmsted; "The Bicycle in Medicine," Dr. C. S. Butler; "The Law," Seward A. Simons; "The Graduating Class," Dr. J. J. Mooney.

